# The Chronic Indigence of Our Hospitals, with Some Suggestions for Its Remedy

**Published:** 1892-04-09

**Authors:** B. Burford Rawlings


### The Institutional Workshop.

### THE CHRONIC INDIGENCE OF OUR HOSPITALS, WITH SOME SUGGESTIONS FOR ITS REMEDY.

By B. Burford Rawlings.

### II.—DIFFICULTIES OF HOSPITAL FINANCE.

When examining the matter of hospital indigence we must remember that beyond the difficulties arising from want of money, there are others in respect of management which assert themselves in the precise ratio of a dependence upon casual gifts. In all their doings, managers must ask themselves how far their contemplated action will obtain or hinder pecuniary help. It is possible that when rules are acted upon impartially, the result is, upon the whole, advantageous, and there is just that much comfort for the courageous, but a beggar who exhibits an independent attitude successfully must be endowed with rare gifts of discretion, and most men would find it almost impossible to remove their actions beyond the influence of their exigencies. A hospital with sufficient means would have no excuse for recognising expediency as a power; with an impecunious hospital expediency is all-powerful. "If anything is done which I disapprove of, or omitted which I desire, my subscription will be withdrawn." These are the conditions accompanying some contributions, and they make themselves felt in almost every detail of the work. A gentleman, well-known for his munificence in certain quarters, withdrew his support from a hospital because a particular method of treatment he advocated was not adopted in respect of a patient admitted at his recommendation; while another threatened secession because the managers had accepted the proceeds of a dramatic performance. A third remonstrated indignantly because the collector's circular informed him that his subscription was "due," for how, he asked, with unconscious irony, "can any voluntary offering be 'due'"? A fourth remarked that it is an "impertinence" to ask for arrears of subscriptions. "Please erase my name," wrote a lady, "for I will give to no institution which wastes its money upon sealing-wax." "Why do you not send all your circulars open and so save postage"? is one enquiry. "I consider it an impertinence to address me upon a post-card," came as a prompt corrective of this economical proposal. "I wish you would take steps to prevent my being so often troubled for tickets," is the aspiration of not a few contributors; while "I see no use in continuing my subscription, because I have so few applications for letters," is the substance of not unfrequent announcements.

If poverty be demoralising, the process affects the victim rather less, perhaps, than those he is brought into contact with. A poor man is a nonentity, and must be made to feel he is. This attitude precisely is adopted in dealing with indigent institutions. People will object to everything which tands in the way of their own wishes. Not one enquirer in twenty will approve a plain, businesslike invitation to subscribe. Although the suggested subscription probably represents but a fraction of the cost of treating the patient to be nominated, it must be begged for as a favour. Where, we may ask, except in indigent hospitals, do reasonable people expect to obtain goods without payment? A popular notion of a charitable institution is a place where the door is always open, and everybody who enters is respectfully requested to help himself to whatever he fancies. It shows something of the helplessness of indigent institutions that aid is occasionally withdrawn for no better reason than that an official has blundered. A failure on the part of a hall-porter to recognise the importance of a visitor has lost friends to an institution, while some people never forgive an undetected printer's error. Many years ago there was a gentleman who subscribed to a particular hospital; his name was Mudge, his Christian name began with an S, and he enjoyed some reputation as an amateur artist. His name, after being entered upon the books as S. Mudge, Esq., was, by an unfortunate inspiration of the printers, altered to Smudge, Esq., and so reached him. As a consequence, the hospital lost his guinea and, as was reported, incurred other losses beside by reason of his never appeased indignation.

Then with regard to raising funds. How many people are practically acquainted with the mean details comprehended in the issue of an appeal? The initial direction in the old cookery-book comes aptly. "First catch your hare," and those who contemplate sending forth an appeal must begin a similar quest. To the inexperienced, the directory appears to offer a prolific field, but it is delusive. Calculations as accurate as circumstances allow, show that only one person in a hundred subscribes to hospitals, and for individual institutions to address appeals to the population as a whole, is an entirely futile proceeding. It is those who have already given who must be invited to give again, and as it is certain every one of this number has been already subjected to the solicitations of numerous other societies, what wonder that the results are scarcely commensurate with the labour? A single substantial contribution in return for every two hundred appeals issued is something to be thankful for, and were it not that nearly every institution is cherished by an infinitesimally small group of ultra-generous friends, what may be asked, would become of the system of support by voluntary contributions?

The statistics referring to the erection of a certain hospital show that as the result of several so-called public meetings, from which the public consistently stayed away, a dinner, and the issue through post of some 50,000 appeals, the sum of £55,287 was raised. Towards this total, 25 persons, most of them privately approached, contributed £36,295, and 88 persons, inclusive of the 25, contributed £45,893. Towards the maintenance of the same hospital a sum of £10,302 was contributed in donations during three successive years, and of this, two thirds were supplied by no more than 36 persons.

Now and again people make public their objections to appeals, and use harsh expressions in regard to them. If we acquit these writers of a desire to injure the work pleaded for, when they urge the receivers of appeals to consign them to the flames, to put them in the waste-paper basket, to return them unstamped to the sender—to do anything rather than respond, they must be charged with prodigious ignorance of their subject. Not one has shown the ability to suggest an alternative, unless it be to do nothing. They write confidently about the needlessness and waste of appeals, just as though the managers of hospitals issued them in sheer wantonness of spirit, negligent that all they need have is forthcoming without being asked for, but to this pleasant theory every worker will oppose the irresistible logic of fact.

Do these writers happen to know anything of hospital finance except by hearsay? Could they estimate, for instance, the thought, labour, and expenditure necessary to the collection of the £100,000 not long ago raised by Guy's Hospital, notwithstanding the princely gifts of a few generous men, and the warm and merited advocacy of a powerful press? Do they know that there is scarcely a hospital in London which could trust to unsolicited help or dispense with the benefactions of friends, and that for months together the receipts from voluntary sources of some of our best known and most deserving institutions fail to cover the expenditure of a single week? If they have no knowledge, and are not prepared with an alternative, their interference seems little justified, although it is true that they succeed in rendering the financial task harder than it need to be. Because these writers object to appeals, appeals must be multiplied, importunity must grow more importunate, and greater sums must be lavished upon the machinery of begging.

As things are, there is but one alternative to importunity, and that is to lessen the work. A hospital, no doubt, would be fully justified if it decided to do no more than the means supplied are sufficient for. What legal, or for that matter, what moral obligation is there upon a hospital committee and its fellow workers to be for ever straining their energies to extend and perfect a work undertaken in the interests of the community, but about which the community is indifferent? Of course, the authorities are bound to make the utmost of the charity's actual resources, but there can be no responsibility beyond that—in regard to what has no present being. A hospital with 100 beds cannot be required to provide 200 beds merely because there are 200 suffering fellow creatures who need admission. Why should it matter to the Hospital Committee more than to any other body of persons that these 100 sufferers are denied tendance? Yet, happily for the sufferers, it is more than likely the authorities of our hypothetical hospital take a different view, and possibly the 100 additional beds are provided. When this is accomplished, the chances are we shall find that a large proportion of these beds will be filled by the nominees of the very economists, organisers, censors, and critics generally who have refused the authorities not only their assistance but their sympathy. Take, for instance, the C.O.S., whose impersonality allows it to be introduced here by way of illustration. Its love for hospitals is purely predatory. It is offended by their methods, it reprobates their excesses, and it never subscribes to their funds, but it advertises for hospital "letters," and it has perfected a system of utilising the hospitals for its nominees. Now, supposing the hospitals to decide upon doing no more than their reliable incomes could pay for, what form would criticism take then? There appears to be nothing to prevent the supporters of a voluntary hospital from shutting its doors, just as they now close a ward.

Again, hospitals in their desperate efforts to attract funds, commit themselves in almost reckless fashion to heavy obligations in the future. Happily, the most liberal contributors are content to help forward the general cause without insistance upon individual privileges, but among minor reforms it might be enacted that hospital letters, if retained at all, should be available only for the poor of the district in which the subscriber lives, unless upon the payment by the real nominator of a substantial registration fee, not with a view to interpose artificial difficulties for the patient to surmount—it would be well if there were none —but to ensure something approaching reciprocity on the part of localities benefited. It is nothing uncommon for patients to attend with letters which have travelled over half the kingdom before being finally appropriated. But letters are clumsy contrivances at best, and it is open to argument whether, as at present disposed of, they do not bring about a result the exact opposite of what is intended, inasmuch as anyone who has obtained a letter feels no sort of compunction about using it, and would be greatly surprised were it suggested that it is not a full equivalent for the advantages it procures. It almost appears as though some people persuaded themselves that the pieces of paper were possessed of a magical power, or at least were charged with the value of the treatment, food, and nursing of a patient apiece. Subscribers of small sums will often forget that many subscriptions are required to cover the cost of a single patient, and the request for the payment of an expected subscription is sometimes refused "because I have no patient to send at present."

The system of privileges affords many opportuniteis for well-to-do people to make use of the contributions of others. It is a fact that if only those non-subscribers who send patients could be brought to contribute a substantial portion of the cost of their nominees, much would be done towards enabling the hospitals to pay their way. In a particular institution there were received within three or four weeks upwards of fifty requests from non-subscribers for the admission of servants, poor neighbours, and others in whom they were "interested." The suggestion to subscribe was openly resented by a few, received in silence by a large majority, evaded by some half-dozen, and acted upon by two. A case of evasion may be mentioned which involves one of our greatest railway companies, and it is typical, because powerful organisations of this kind and large employers of labour are among the most ready to make use of our hospitals without doing anything in return. Application was made from a large local station for the admission of a signalman who had been in the employ of the company for seventeen years. Naturally, a subscription was suggested. No reply was made, but after a few days' interval the medical man in attendance began a correspondence. Again the question of a subscription was raised. A demand followed from the General Manager in London for a list of subscribers, and a third suggestion as to the subscription was received in silence. Nothing more was heard of the case until a poor man attended at the introduction of one of the hospital staff who had been approached by a friend in London, who in his turn had been written to by the local doctor, with the result that an appointment had been unwittingly made for the very man the Company was interested in, and who was found to be so ill that admission could not be refused. That patient, the nominee and dependent of a company whose authorities, out of a gross weekly revenue of some £150,000, have not seen their way to send a single guinea to the hospital they did not scruple to make use of, has already been in the wards some sixteen weeks, a charge upon an institution maintained by voluntary subscriptions, surely not contributed in order that a wealthy corporation might evade an obvious duty, but the performance of which the hospital authorities are absolutely powerless to enforce. The attempt to compel a subscription would merely bring punishment upon the unhappy and altogether innocent patient.

No doubt one duty of every prosperous citizen is to accept his share of the cost of restoring the sick poor to health. But then it is equally incumbent upon him to help maintain the State and the police, and in these cases it has not been found either safe or desirable to leave his assessment to his own unaided judgment. This indicates one of the difficulties inseparable from a purely voluntary method. Reasons why people should not contribute to any given object are always to be found if sedulously sought for. Many people, for instance, think they dispose of the claims of the hospitals by attributing improvidence to the patients. The charge may be true in a vast number of cases, but if so, are we prepared to pass a death sentence? That this matter of self-help should be urged upon all who receive hospital relief is undeniable, and an endeavour will be made presently to show how large a total patients' contributions might attain to under enlightened conditions. Hospitals are, however, necessities, not to one class, but to all. The strictly logical outcome of this is that their maintenance should be provided for by a general levy, and if we reject this conclusion, it is because the desire to preserve a philanthropic character to our hospitals is a sufficient reason for sacrificing consistency. The lowering of the status of the managing bodies to the level of guardians would be nothing short of a calamity. Under parochial government, the lamp of science would grow dim; philanthropy would expire, and the hospitals would deteriorate into mere asylums, unwarmed by a spark of enthusiasm, and unlighted by one ray of progress. It is possible that the same objections would apply in less degree to an Imperial subsidy, especially if it took the form of a premium, to be distributed by an authority largely composed of hospital representatives; but looking to all the circumstances, we must conclude that State support, however carefully and covertly rendered, would necessarily involve some sort of State control, and is best avoided, until at least all other means have been exhausted.

